# CLRM-05. DRUG-RELEASING MICRODEVICES TO PREDICT RESPONSES TO TARGETED THERAPIES IN PATIENTS WITH GLIOMAS

**DOI:** 10.1093/noajnl/vdab112.004

**Published:** 2021-09-21

**Authors:** Sarah Blitz, Christine Dominas, Michael J Pannell, E Antonio Chiocca, Patrick Y Wen, Oliver Jonas, Pierpaolo Peruzzi

**Affiliations:** 1 Harvard Medical School, Boston, MA, USA; 2 Brigham and Women's Hospital, Boston, MA, USA; 3 Dana-Farber Cancer Institute, Boston, MA, USA

## Abstract

Genomic studies of tumor specimens are becoming standard of care in patients with gliomas to characterize druggable molecular features. Unfortunately, with the exception of IDH1-R132 mutation and MGMT promoter methylation, molecular markers have failed to predict clinical responses to drugs, and the impact of targeted therapies remains minimal. There is a need for a high-throughput, patient-specific, and significantly predictive method to inform a most effective personalized therapy. This pilot trial tests the safety and feasibility of drug-releasing microdevices which are temporarily implanted into the tumor during a standard craniotomy. They release microdoses of up to 20 drugs or drug combinations into surrounding tissue in a controlled spatial distribution. The devices, together with a cuff of surrounding tumor tissue, are removed at the end of surgery, and the tissue is analyzed for biological and molecular response markers allowing for in situ characterization of the drug efficacy. Four patients have been enrolled to date, out of a total planned of six. Two microdevices were implanted into each tumor (8 total devices). Average indwelling time in tumor tissue was 139 minutes. Eight devices (100%) were successfully retrieved, and all surgeries were completed without immediate (<24 hours) or delayed (<30 days) complications. Seven (87%) specimens were of adequate quality, allowing for planned histological and molecular studies. For all analyzed specimens, the intraoperative incubation time was sufficient to observe: 1) Drug concentration gradients; 2) Differential molecular signs of cell toxicity (DNA damage and Caspase 3 activation); 3) Whole genome transcriptional changes; 4) Tumor microenvironment composition; and 5) Preliminary evidence of concordance between the biological readout obtained from microdevice analysis and clinical response. Drug-releasing microdevices were well tolerated, seamlessly integrated in standard craniotomy workflow, and allowed for collection of a significant amount of data related to the differential efficacy of multiple drugs in a personalized manner.

